# Gp85 protein encapsulated by alginate-chitosan composite microspheres induced strong immunogenicity against avian leukosis virus in chicken

**DOI:** 10.3389/fvets.2024.1374923

**Published:** 2024-05-22

**Authors:** Tianyu Lei, Rongchang Liu, Liyun Zhuang, Tingting Dai, Qingfu Meng, Xiaodong Zhang, Yinli Bao, Cuiqin Huang, Weiming Lin, Yu Huang, Xintian Zheng

**Affiliations:** ^1^College of Life Sciences, Longyan University, Longyan, China; ^2^College of Animal Sciences, Fujian Agriculture and Forestry University, Fuzhou, China; ^3^Institute of Animal Husbandry and Veterinary Medicine, Fujian Academy of Agricultural Sciences, Fuzhou, China; ^4^Fujian Provincial Key Laboratory of Preventive Veterinary Medicine and Veterinary Biotechnology, Longyan, China

**Keywords:** avian leukosis virus subgroup J, Gp85 protein, alginate-chitosan composite microspheres, vaccine, immunogenicity

## Abstract

**Introduction:**

Avian leukosis, a viral disease affecting birds such as chickens, presents significant challenges in poultry farming due to tumor formation, decreased egg production, and increased mortality. Despite the absence of a commercial vaccine, avian leukosis virus (ALV) infections have been extensively documented, resulting in substantial economic losses in the poultry industry. This study aimed to develop alginate-chitosan composite microspheres loaded with ALV-J Gp85 protein (referred to as aCHP-gp85) as a potential vaccine candidate.

**Methods:**

Sodium alginate and chitosan were utilized as encapsulating materials, with the ALV-J Gp85 protein serving as the active ingredient. The study involved 45 specific pathogen-free (SPF) chickens to evaluate the immunological effectiveness of aCHP-gp85 compared to a traditional Freund adjuvant-gp85 vaccine (Freund-gp85). Two rounds of vaccination were administered, and antibody levels, mRNA expression of immune markers, splenic lymphocyte proliferation, and immune response were assessed. An animal challenge experiment was conducted to evaluate the vaccine’s efficacy in reducing ALV-J virus presence and improving clinical conditions.

**Results:**

The results demonstrated that aCHP-gp85 induced a significant and sustained increase in antibody levels compared to Freund-gp85, with the elevated response lasting for 84 days. Furthermore, aCHP-gp85 significantly upregulated mRNA expression levels of key immune markers, notably TNF-α and IFN-γ. The application of ALV-J Gp85 protein within the aCHP-gp85 group led to a significant increase in splenic lymphocyte proliferation and immune response. In the animal challenge experiment, aCHP-gp85 effectively reduced ALV-J virus presence and improved clinical conditions compared to other groups, with no significant pathological changes observed.

**Discussion:**

The findings suggest that aCHP-gp85 elicits a strong and prolonged immune response compared to Freund-gp85, indicating its potential as an innovative ALV-J vaccine candidate. These results provide valuable insights for addressing avian leukosis in the poultry industry, both academically and practically.

## 1 Introduction

The avian leukosis virus (ALV) is a retrovirus belonging to the *Retroviridae* family, within the *orthoreovirus* subfamily genus (1). It represents a significant threat to poultry production, capable of spreading both vertically and horizontally, leading to adverse outcomes such as tumor growth, immunosuppression, reduced performance, and death in chickens (2). ALV includes seven subgroups, labeled A, B, C, D, J, and K, and also encompasses the endogenous AIV subtype E (3). The economic impact of avian leukosis on the poultry industry is significant, and despite extensive research efforts, a definitive prevention or treatment solution has yet to be found. The elimination of ALV-J from breeding stocks is a challenging and costly process, often limited by technical challenges during batch testing. Consequently, vaccination has become a key strategy for controlling ALV infections. Traditional vaccines, including attenuated and inactivated ones, have been ineffective against ALV (4), leading to the exploration of subunit vaccines as a more viable option. In the quest for effective ALV-J subunit vaccines, the use of adjuvants has shown great promise in enhancing the immune protective effect. Various adjuvants, such as liposomes (5) and carbon quantum dots (CQD) (6, 7), have been utilized to improve the immune response to ALV-J.

In this study, we used alginate-chitosan composite microspheres (aCHP) (8) as a novel adjuvant to boost the effectiveness of the Gp85-based ALV-J subunit vaccine. These biodegradable microspheres improve safety and stability. Chitosan is recognized for its tolerability and potent immune-stimulating properties (9), making it a viable adjuvant candidate. Additionally, these microspheres have the potential to function as a controlled-release adjuvant system similar to polymeric microparticle adjuvants (10). They are effective in encapsulating antigens and act as a delivery mechanism for vaccines, a method proven successful in human and animal vaccinations. The use of alginate-chitosan microspheres for antigen delivery (11–13) is particularly promising. Once administered, they demonstrate excellent safety, high biocompatibility, and a controlled, sustained antigen release (14).

The goal of this research is to evaluate the effectiveness of alginate-chitosan composite microspheres in conjunction with the ALV-J subunit vaccine compared to Freund’s adjuvant. This study addresses the urgent need to improve the efficacy of ALV-J subunit vaccines, offering a potential method for controlling ALV infection. By exploring the capabilities of alginate-chitosan microspheres as adjuvants, this research aims to advance the development of more effective and safer vaccines against avian leukosis, thus helping to reduce economic losses in the poultry industry and enhance poultry health and productivity.

## 2 Materials and methods

### 2.1 Virus strains

The ALV-J strain LY2021J, with GenBank accession number OP918846, was isolated and identified by the Fujian Provincial Key Laboratory of Preventive Veterinary Medicine and Veterinary Biotechnology.

### 2.2 Animals

A total of 60 SPF chicken embryos and 45 one-day-old chicks were acquired from Boehringer Ingelheim company in Jiangsu, China, for the incubation process. This animal study was conducted following the experimental animal guidelines set by the Ministry of Science and Technology in Beijing, China, and received approval from the Ethics Committee of Longyan University (License No: LY2021009x).

### 2.3 Expression and identification of the gp85 protein

The gp85 gene was amplified from the ALV-J strain LY2021J using specific primers (Forward: 5′-ATGTTGCAACAACCAGGAAACG-3′; Reverse: 5′-GCTTCGGTGGTGACCCGT-3′). Subsequently, the amplified fragment was ligated into the pET-28a (+) vector (Takara, China) through ligation, resulting in the recombinant plasmid pET-28a-gp85. Successful cloning was confirmed by PCR amplification with T7-specific primers and pET-28a-gp85 as the template. Agarose gel electrophoresis was used to analyze the PCR products, selecting plasmids with the expected PCR bands for sequencing.

For protein expression, the recombinant plasmid pET-28a-gp85 was introduced into BL21 (DE3) *E. coli* cells. After culturing, the cells were lysed, and the precipitated proteins were collected. To purify the gp85 protein, the proteins were denatured in 8 M urea and then purified using column chromatography. Additional purification was performed through dialysis at 4°C, using dialysis bags with suitable molecular weight cutoffs (MWCO: 8000–14,000, United States). The quality and integrity of the purified protein were verified using sodium dodecyl sulfate-polyacrylamide gel electrophoresis (SDS-PAGE) and protein blotting, with the help of anti-ALV-J gp85 G2 antibodies (Kerafast, United States). The concentration of the purified protein was measured using a BCA Protein Assay Kit (Sangon Biotech, China).

### 2.4 Preparation of gp85 protein sodium alginate-chitosan composite microspheres

The alginate-chitosan composite microspheres (aCHP) were prepared using an emulsification crosslinking method, slightly modified from a standard protocol. Briefly, 8.8 mL of highly purified recombinant Gp85 protein (200 mg/mL in pH 8.0 PBS) was mixed with 1.2 mL of Tween80 (Sangon Biotech, China) and 10 mL of a 2.0% alginate solution. The mixture was stirred gently with a magnetic bar for 10 min. Next, 30 mL of liquid paraffin (Sangon Biotech, China) and 1.2 mL of Span 80 (Sangon Biotech, China) were added and stirred at 800 rpm for 10 min. Then, 10 mL of a 2% (w/v) CaCl_2_ solution was slowly added to the emulsion and stirred at 1,200 rpm for 30 min to induce cross-linking and solidification.

After centrifugation at 1,500 g for 10 min, the pellet was collected and washed three times with 0.1 mol/L sodium acetate buffer to obtain the alginate-encapsulated microspheres. Then, 5 mL of microsphere suspension was mixed with 20 mL of chitosan solution (0.5% concentration, pH 4), stirring at 1,500 rpm for 20 min. After another centrifugation at 1,200 g for 10 min, the pellet was collected and washed three more times with 0.1 mol/L sodium acetate buffer to prepare the alginate-chitosan encapsulated microspheres.

### 2.5 Microspheres morphology observation and particle size determination

Microsphere morphology was examined using scanning electron microscopy (SEM; Hitachi, S3400 N, Japan). The microspheres were prepared in sodium acetate buffer and coated with a thick layer of conductive platinum using a sputter ion coater. SEM images were taken at a 15 mm working distance with a 15 keV beam. Particle size distribution was analyzed using a particle size analyzer (Microtrac, S3500, United States), with images processed using Microtrac-FLEX software.

### 2.6 Determination of drug encapsulation efficiency and drug loading capacity of microspheres

The microspheres’ encapsulation effectiveness was assessed using a previously established extraction method (15). In this method, 1 mL of 1 M NaOH (pH 11.5) was added to a tube containing 10 mg of alginate-chitosan microspheres with Gp85, and the tube was rotated at 30 rpm for 12 h at 37°C. Afterward, the samples were centrifuged, and the supernatant was used to measure antigen content using a BCA protein assay kit (Beyotime Biotech, China). Encapsulation efficiency (EE) was calculated using the following formula: encapsulation efficiency (EE) = (amount of protein in solution − amount of protein in supernatant)/amount of protein in solution × 100%. The loading capacity (LC) was determined using the following equation: Drug loading capacity = by (total mass of protein antigen added − mass of antigen in supernatant)/mass of dried microspheres × 100%.

### 2.7 *In vitro* drug release assay

The microspheres were incubated in 25 mL of 0.2 mol/L PBS buffer (pH 7.4) at 37°C and stirred at 120 rpm for 24 h. To quantify the antigen concentration in the release medium, 0.5 mL samples were extracted at 2 h intervals, with an equivalent volume of fresh release solution was replenished (8). A cumulative release curve of the antigen from the microspheres was plotted, using the release time as the horizontal axis and the cumulative release rate as the vertical axis.

### 2.8 Preparation of Freund’s adjuvant Gp85 subunit vaccine

For comparison, a vaccine was prepared using Freund’s incomplete adjuvant (FIA) as a standard adjuvant. This vaccine was formulated by gradually adding 5 mg of Gp85 antigen to 5 mL of PBS, followed by mixing with 5 mL of Freund’s incomplete adjuvant (FIA) (Biosharp, Shanghai, China) at 800 rpm for 30 min to create a water-in-oil emulsion. The emulsion was then stored at 4°C for subsequent use.

### 2.9 Safety test

Following the World Organization for Animal Health guidelines (2012), six 1-day-old specific pathogen-free (SPF) chickens received intramuscular injections for 2 consecutive weeks. The chickens were monitored daily for any signs of local reactions, clinical symptoms, or mortality.

### 2.10 Immunization of chickens

The animal study was conducted according to the experimental animal welfare guidelines issued by the Ministry of Science and Technology, Beijing, China. The study protocol was approved by the Ethics Committee of Longyan University (permit number: LY2021009x), adhering strictly to the ethical standards for laboratory animal care and use.

Forty-five SPF chickens were randomly divided into three groups (*n* = 15 chickens each) as follows:

Group 1 (aCNP-gp85): received gp85 protein sodium alginate-chitosan composite microspheres.
Group 2 (Freund-gp85): received a solution of gp85 adjuvanted with Freund’s adjuvant.
Group 3 (NC): served as the negative control (saline alone).

The immunization schedules, including routes and doses, are detailed in Table 1. Chickens were immunized on days 3 and 15, with weekly venous blood collections. On day 44, spleen lymphocytes were harvested from five randomly selected chickens in each group.

**Table 1:** Immunization and challenge list.

Groups	Vaccine formulations	Antigen dose	Challenged virus
aCHP-gp85	Microsphere loading Gp85 protein	600 μg	10^5^ TCID50/0.5 mL
Freund-gp85	Gp85 protein with Freund’s adjuvant	600 μg	10^5^ TCID50/0.5 mL
NC	PBS	0.5 mL	—

#### 2.10.1 Antibody titer in response to vaccination

To evaluate the humoral immune response, antibody titers against ALV were measured using ALV antibody enzyme-linked immunosorbent assay (ELISA) kits (Ruifan Biotechnology, China), according to the manufacturer’s guidelines. Serum samples from each group were tested in triplicate, and an S/P ratio above 0.6 indicated positive ALV-J antibody presence.

#### 2.10.2 MTT assay of splenic lymphocytes proliferation

In this study, spleen cell suspensions were obtained from chickens through the mincing of spleen tissue and filtration through a fine mesh. The spleen remnants were utilized for RNA extraction. To eliminate erythrocytes, 0.8% ammonium chloride solution was used, followed by rinsing in DMEM. The cells were then counted, suspended in DMEM containing 1% antibiotics and 10% FBS, and adjusted to a concentration of 10^7^ cells/mL. The cell suspensions were added to sterile 96-well round-bottom tissue culture plates, with each chicken’s cells cultured in triplicate wells. Gp85 protein at a concentration of 1 mg/mL was added as a stimulant. The plates were incubated at 37°C and 5% CO_2_ for 72 h. Post incubation, the supernatant was discarded, and MTT solution (0.5 mg/mL) was added to each well for 4 h. After aspirating the MTT solution, DMSO (150 μL/well) was added, and absorbance was measured at 570 nm. The stimulation index (SI) was calculated to assess the immune response, indicating cell proliferation and activation in response to the gp85 protein.

#### 2.10.3 Cytokine analysis

Spleen tissue was utilized for RNA extraction (Vazyme Biotech, Nanjing, China), followed by cDNA synthesis according to the manufacturer’s protocol using the First Strand cDNA Synthesis Kit (Vazyme Biotech, Nanjing, China). Real-time quantitative polymerase chain reaction (RT-PCR) was performed in a 25 μL reaction mixture, including 5 μL of cDNA, 1 μL of both upstream and downstream primers (see Table 2) for IL-1β, IL-2, IL-6, IFN-γ, TNF-α, and GAPDH, and 12.5 μL of 2× Maxima SYBR Green (Vazyme Biotech, Nanjing, China). The CT values were determined using the 2^−ΔΔCCT^ method, which calculated the fold change relative to the expression of the housekeeping gene GAPDH and cytokine genes. The relative quantification was expressed as *R* = 2^−ΔΔCCT^.

**Table 2:** Primers for qPCR.

Segment	Primer	Sequence (5′–3′)
IL-1β	PF	GGTCAACATCGCCACCTACA
	JR	GGTCAACATCGCCACCTACA
IL-2	PF	GCTAATGACTACAGCTTATGGAGCA
	AR	TGGGTCTCAGTTGGTGTGTAGAG
IL-6	PF	AAATCCCTCCTCGCCAATCT
	BR	CCCTCACGGTCTTCTCCATAAA
IFN-γ	PF	GACAAGTCAAAGCCGCACA
	BR	TCAAGTCGTTCATCGGGAGC
TNF-α	PF	TTCTATGACCGCCCAGTT
	BR	CAGAGCATCAACGCAAAA
GAPDH	PF	GGCACTGTCAAGGCTGAGAAC
	BR	AGATGATAACACGCTTAGCACCAC

#### 2.10.4 Challenge experiment

On the 43rd day after the initial immunization, all chickens, except those in the negative control group (NC), received an intraperitoneal injection of 0.5 mL containing 105,105 TCID50 of ALV-J LY2021 virus per chicken. A separate group, designated as the infection control group (NC-Infection, *n* = 10), received injections of the virus suspension at the same dosage. Aseptic plasma samples were collected weekly to monitor viral viremia in the chickens using an ELISA ALV-J antigen detection kit. The chickens were weighed weekly, and humane euthanasia was conducted in the sixth week post-challenge. Organ indices were calculated with the formula: Organ index = (Organ tissue weight/Chicken body weight) × 100%.

Challenge group 1 (aCNP-gp85) received gp85 protein sodium alginate-chitosan composite microspheres.
Challenge group 2 (Freund-gp85) received a solution of gp85 adjuvanted with Freund’s adjuvant.
Challenge group 3 served as the negative control (saline alone).
Challenge group 4 represented NC-Infection.

#### 2.10.5 Viral loads in the organs

To evaluate the viral load in organs following infection with ALV-J, a qPCR method was developed. The PCR products, amplified using specific primers (Forward: 5′-GATGCCTTGCCAAGCCCTGTC-3′, Reverse: 5′-AGATGATAACACGCTTAGCACCAC-3′), were cloned into a pMD19-T vector, forming the recombinant plasmid pMD-ALV. Standard curves were generated by diluting plasmid pMD-ALV in a range from 10^4^ to 10^9^ copies using TE buffer. Real-time PCR was performed using an Eppendorf Mastercycler ep realplex instrument (Eppendorf, Hamburg, Germany) with TB Green^®^ Premix Ex Taq^™^. Total RNA was extracted from the heart, liver, spleen, and kidney to quantify the viral load in terms of copies per milligram of tissue.

#### 2.10.6 Statistical analysis

Statistical analysis was performed using SPSS (Version 11.0, SPSS Inc., Chicago, IL), with all data presented as mean ± standard error. Measurements were recorded in triplicate, and significance was determined at a *p*-value of <0.05. Data visualization was facilitated using Origin software (Version 2018, OriginLab, Northampton, MA).

## 3 Results

### 3.1 Expression and characterization of recombinant Gp85 protein

**Figure 1:**
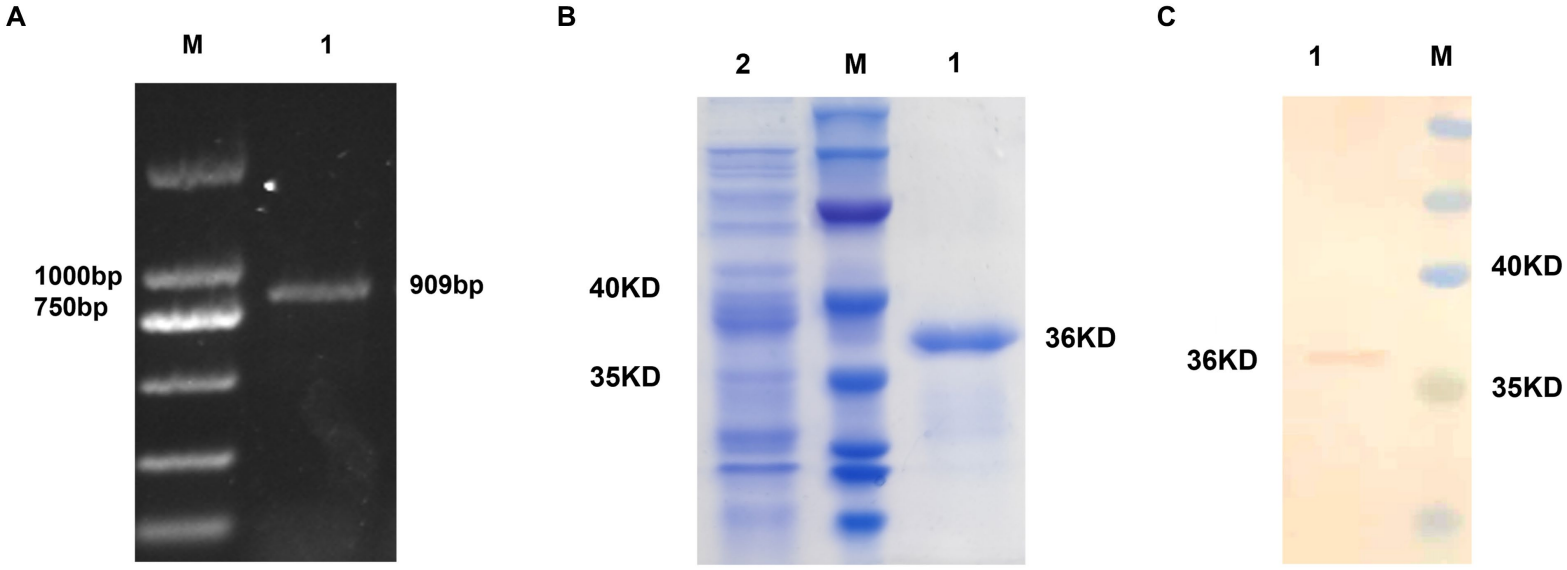
Prokaryotic expression of the gp85 protein. **(A)** Amplification of the gp85 gene by PCR; M: Marker 1: gp85 gene. **(B)** Recombinant gp85 protein detection by SDS-PAGE assay M: Marker 1: gp85 protein 2: Empty plasmid control. **(C)** Recombinant gp85 protein detection by western blot assay M: Marker 1: gp85 protein.

A 909-bp fragment was successfully PCR amplified from ALV-J cDNA, yielding the expected result (Figure 1A). The recombinant plasmid pET-28a-gp85 was used as a template for PCR amplification with T7 primers, followed by sequencing to confirm the correct insertion of the target gene into the vector. SDS-PAGE analysis confirmed the bacterium successfully expressed the desired protein, approximately 36 kD in size (Figure 1B). Protein blot analysis was performed using Anti-ALV-J gp85 G2 antibodies (Figure 1C), and the gp85 protein concentration was determined to be 1 mg/mL using a BCA Protein Assay Kit and thin-layer chromatography scanning.

### 3.2 Characterization of aCHP-gp85

**Figure 2:**
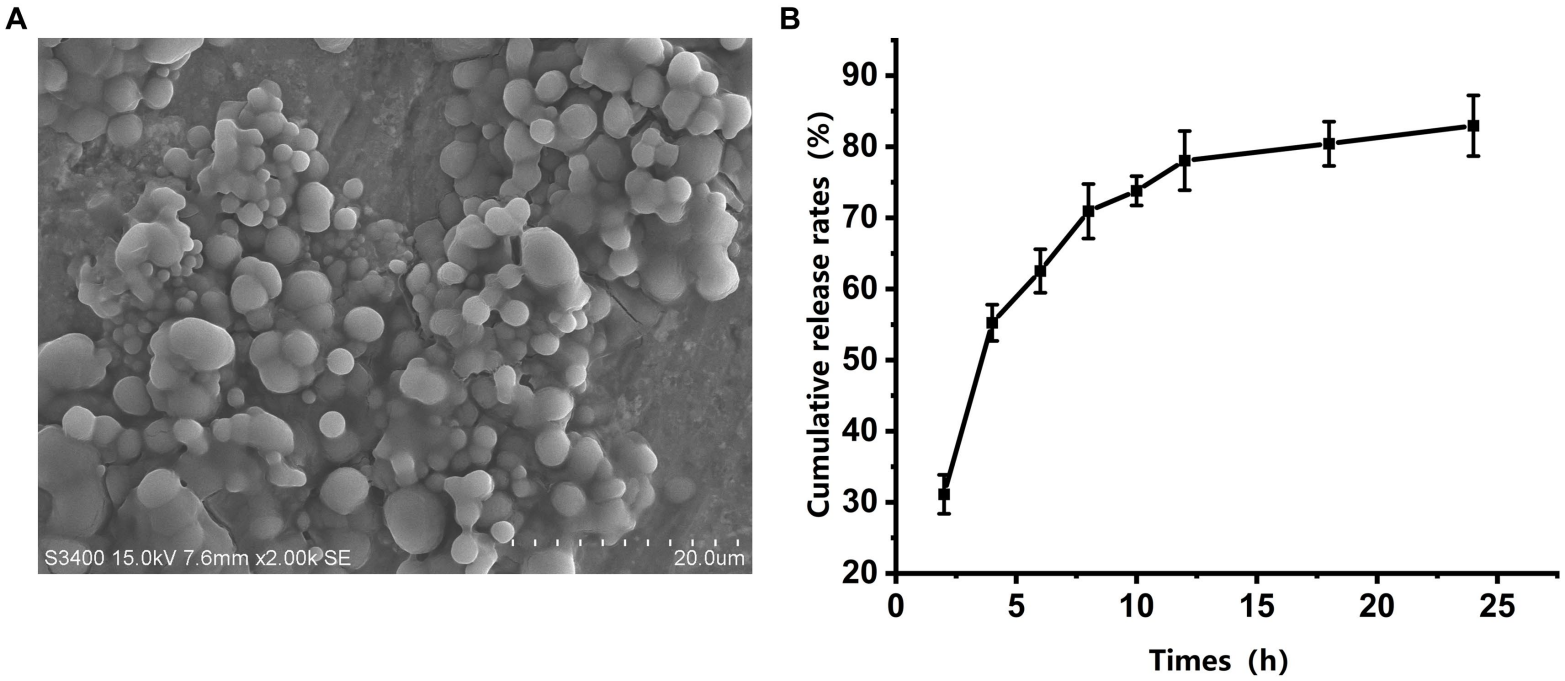
Morphology and *in vitro* release of gp85 from microspheres. **(A)** Representative scanning electron microscopy image of a microsphere. **(B)** *In vitro* cumulative release profiles of gp85 from the microspheres during 24 h, determined using BAC kit and corrected for EE.

Scanning electron microscopy (SEM) revealed that the majority of the microspheres were round and well-dispersed (Figure 2A). The average diameter of 200 microspheres was 2.65 ± 0.28 μm. Using Equations 1 and 2, the antigenic protein content was estimated through the BCA technique. The microspheres had an average encapsulation efficiency of 76.3 ± 0.73% and a drug loading rate of 3.26 ± 0.05%. The release kinetics showed a two-phase release: a rapid release in the first 12 h followed by a slower release, with a total release of 83.36% of the antigen protein within 24 h (Figure 2B).

### 3.3 Detection of ALV-J specific IgG antibody

**Figure 3:**
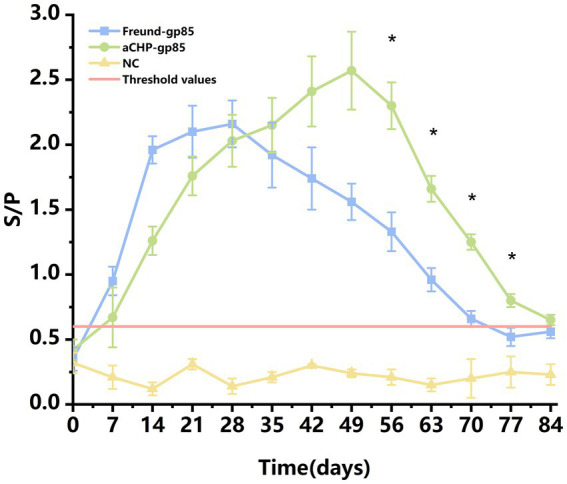
Variation in chicken serum antibodies in response to different vaccines with ALV-J antibody positivity. Changes in the levels of serum antibodies in chicken blood with different vaccines. Serum samples with a *S*/*p*-value higher than 0.6 were considered ALV-J antibody positive.

The efficacy of aCHP-gp85 in eliciting an immune response was assessed by measuring anti-ALV gp85 antibody levels in 15 chickens from each group (Figure 3). Both the aCHP-gp85 and Freund-gp85 groups successfully induced anti-ALV-J antibodies. The Freund-gp85 group showed increasing immunoglobulin levels up to 28 days post-immunization, whereas the aCHP-gp85 group showed an increase up to 49 days, peaking at an S/P level of 2.57 ± 0.29. Antibody responses were maintained for over 63 days, with significantly higher levels in the aCHP-gp85 group from day 56 to 77 (*p* < 0.05). The IgG retention time was also longer in the aCHP-gp85 group (84 days) compared to the Freund-gp85 group (77 days). The aCHP-gp85 group achieved a 100% antibody positivity rate using the ALV-J antibody kit threshold (S/*p* > 0.6).

### 3.4 Effect of Gp85 protein on the proliferation of splenocytes in immunized chickens

**Figure 4:**
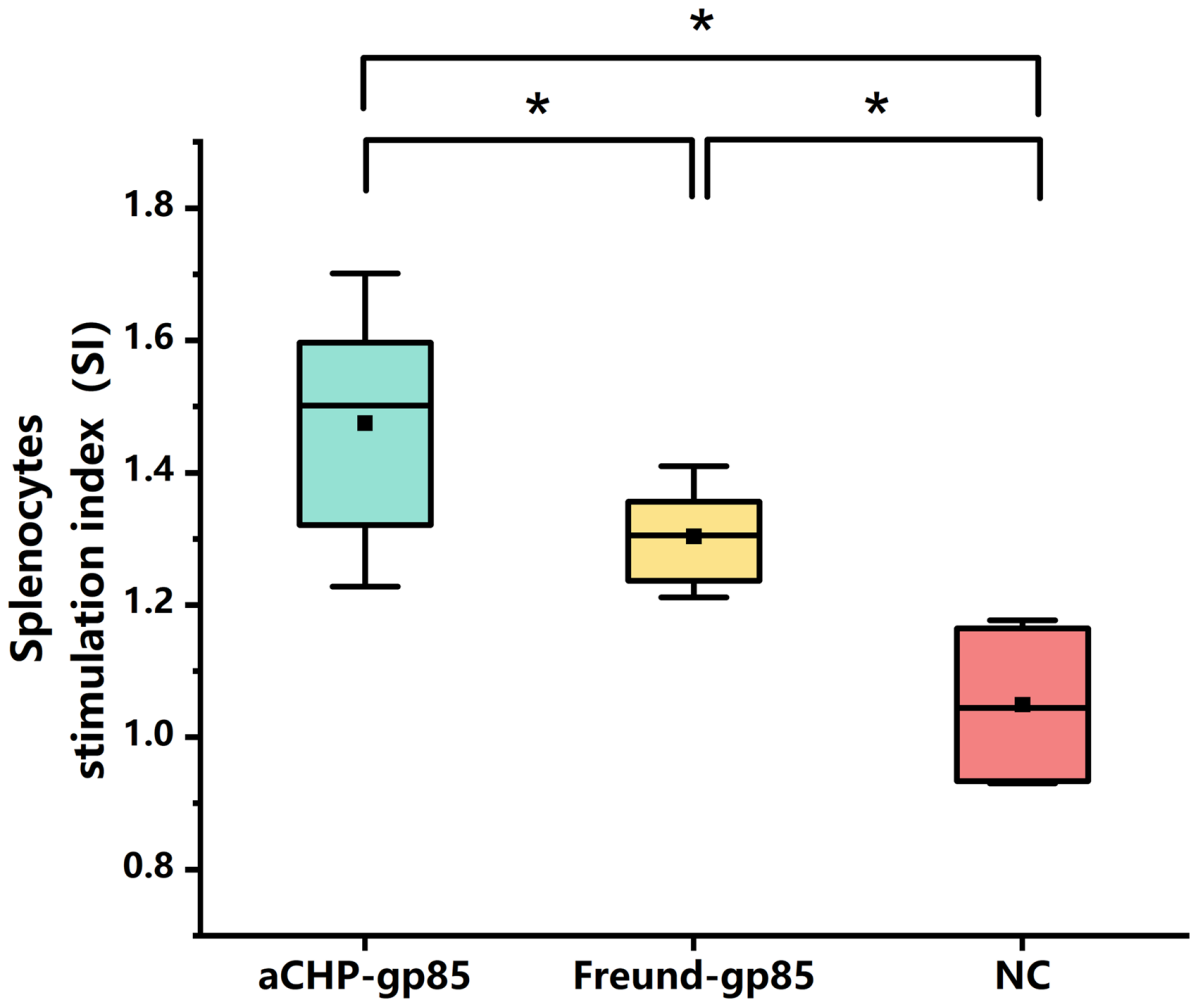
Evaluation of lymphoproliferation response to gp85 protein 43 days after group vaccination with various Gp85 protein formulations. Lymphoproliferation response to Gp85 protein 43 days after group vaccination with different gp85 protein formulations. Results for each group represent mean stimulation index (SI) ± SD of 3 chicken per group from 3 separate experiments. Groups with different letters indicate statistically significant difference (^*^indicates *p* < 0.05 and ^**^indicates *p* < 0.01).

The lymphocyte proliferation test (splenic lymphocyte stimulation response) was carried out 43 days post-inoculation. Five chickens from each group were chosen for aseptic spleen extraction. The spleen cells from each chicken were isolated, activated with the gp85 protein, and assessed for proliferation. The results showed that the proliferative responses in both the aCHP-gp85 and Freund-gp85 groups were significantly higher than those in the normal control group (*p* < 0.05), with the aCHP-gp85 group demonstrating superior spleen lymphocyte proliferation compared to the Freund-gp85 group (Figure 4).

### 3.5 Cytokine expression analysis

**Figure 5:**
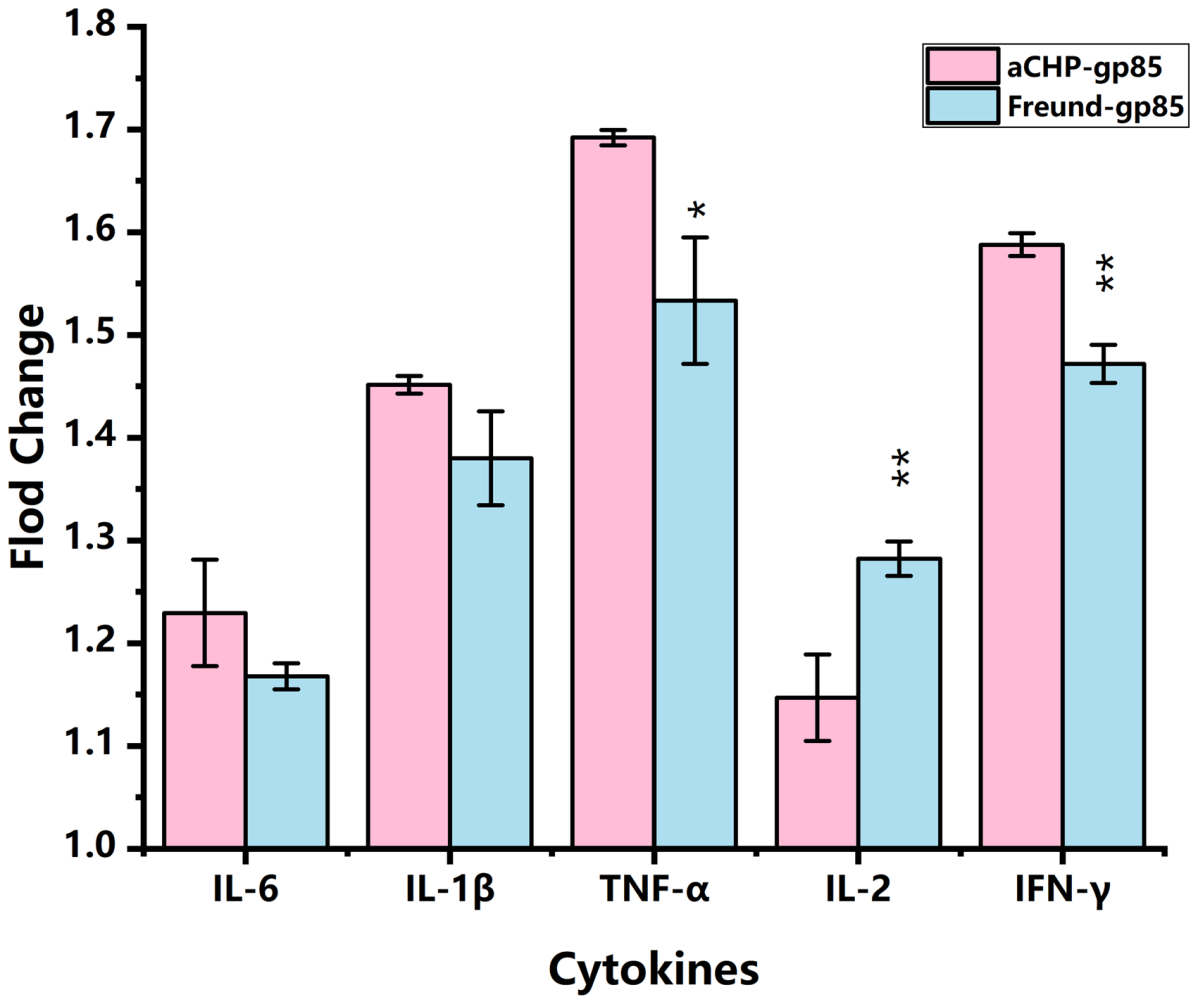
Comparison of cytokine transcript levels in each group relative to the negative control (NC). Transcript levels of cytokines in each group relative to the negative control (NC) group (^*^indicates *p* < 0.05 and ^**^indicates *p* < 0.01).

Figure 5 illustrates that mRNA transcript levels of IL-6, IL-1β, TNF-α, IL-2, and IFN-γ were upregulated in both the aCHP-gp85 and Freund-gp85 groups compared to the NC group. Notably, the levels of TNF-α (p < 0.05) and IFN-γ (*p* < 0.01) mRNA transcripts were significantly higher in the aCHP-gp85 group than in the Freund-gp85 group. However, IL-2 transcript levels were significantly lower in the aCHP-gp85 group (p < 0.01).

### 3.6 Protective efficacy of the aCHP-gp85

#### 3.6.1 Clinical findings

**Figure 6:**
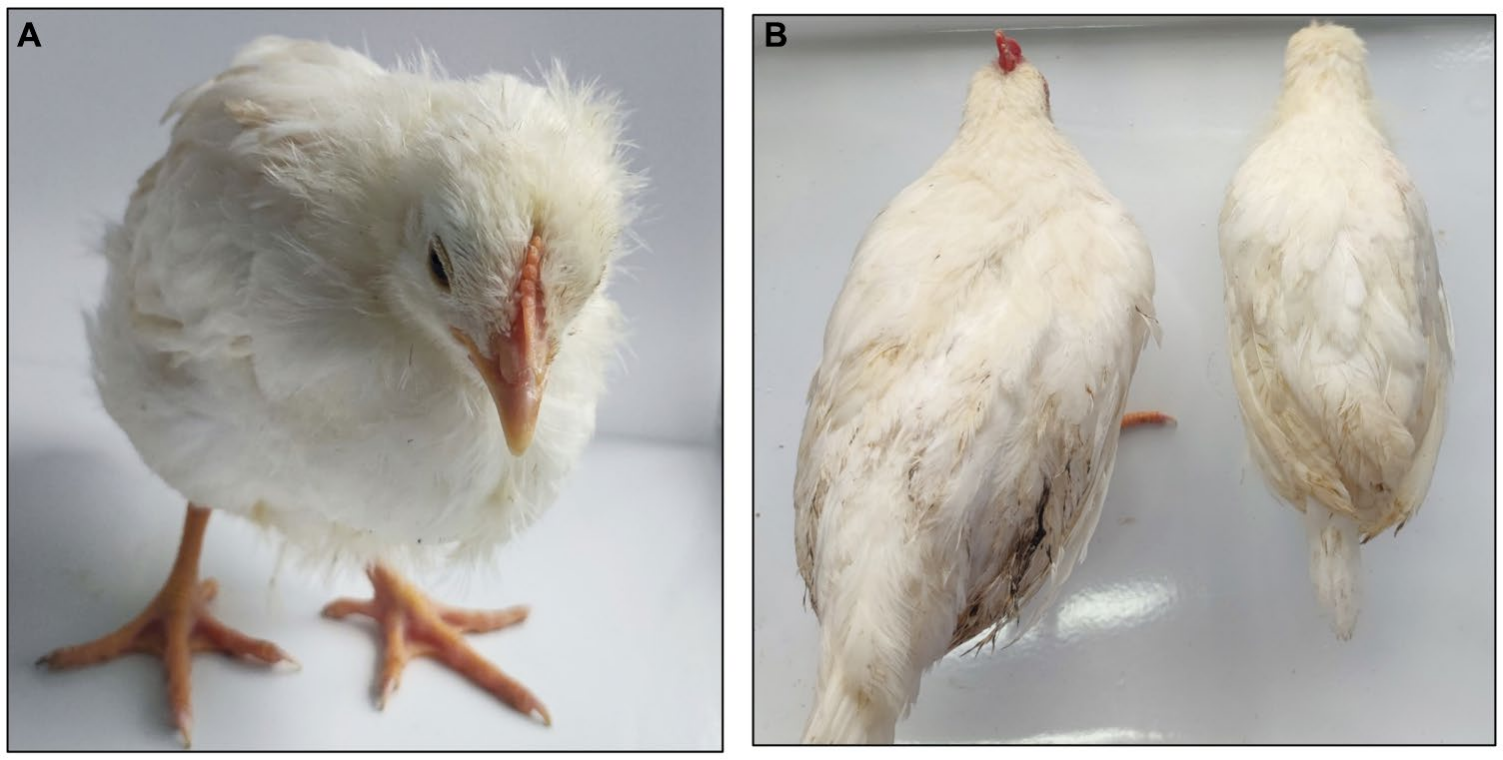
Comparing symptoms in chickens. **(A)** NC-infections group exhibited depression, wrinkled feathers, and sleepiness. **(B)** Chickens in the aCHP-gp85 group (left) and the chickens in NC-Infections (right) showed significant dysplasia.

Throughout the monitoring period, chickens in the aCHP-gp85 and NC groups showed no clinical symptoms. In contrast, chickens in the NC-Infections group exhibited signs of distress, including reduced feed intake, stunted growth, ruffled feathers, and lethargy, as shown in Figure 6.

#### 3.6.2 Viraemia test results

**Figure 7:**
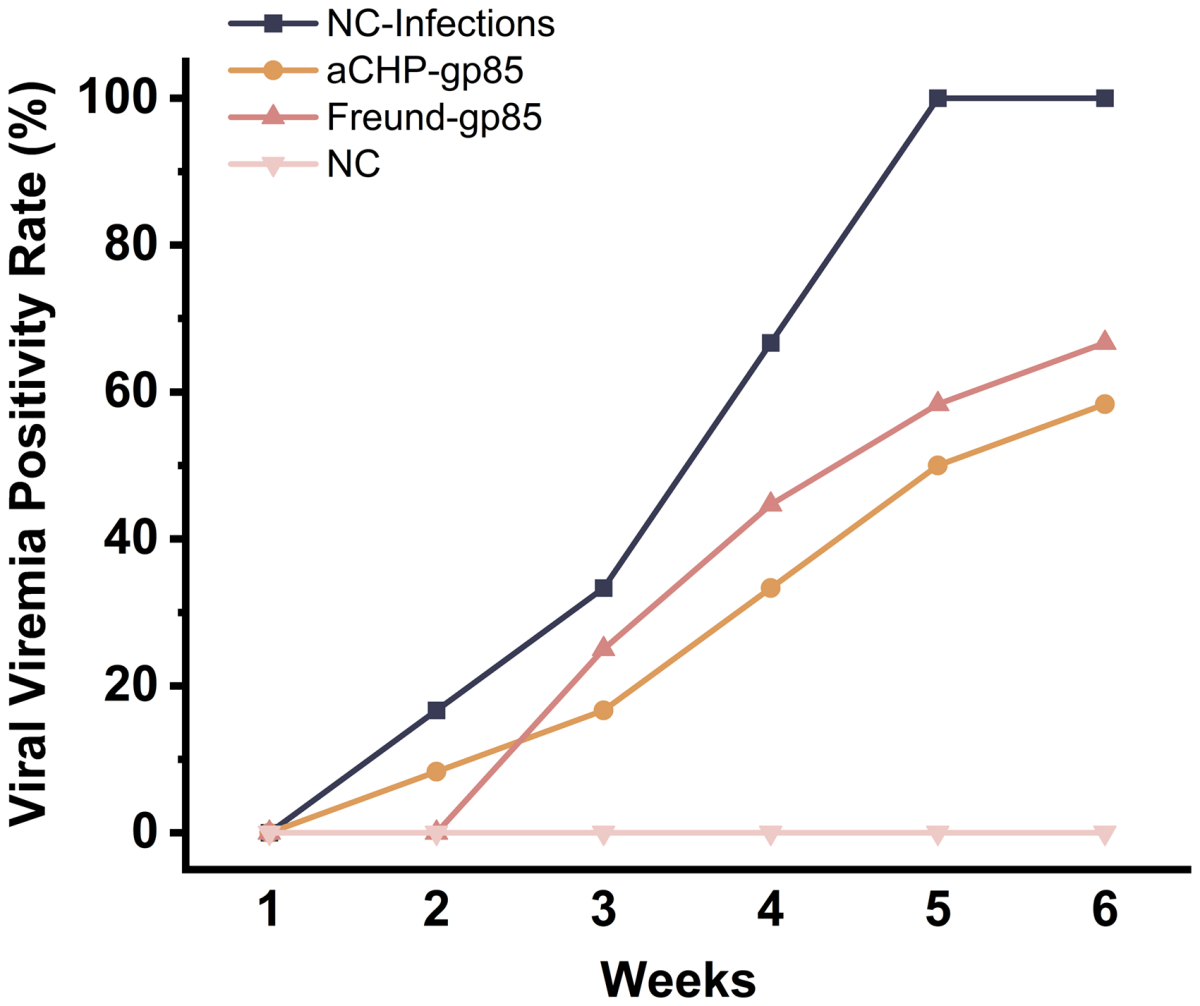
Positivity rate of ALV-J viremia in each group. The viral viremia positivity rate of chicken in each group post-challenge.

Chickens from each group were tested for viremia at weekly intervals after exposure, as detailed in Figure 7. The aCHP-gp85 and Freund-gp85 groups had lower rates of positive viremia compared to the NC-Infections group, with the aCHP-gp85 group showing the lowest rates. This suggests that the aCHP-gp85 and Freund-gp85 vaccines were effective in reducing infection rates and providing protection, with aCHP-gp85 being notably superior.

#### 3.6.3 Autopsy results

**Figure 8:**
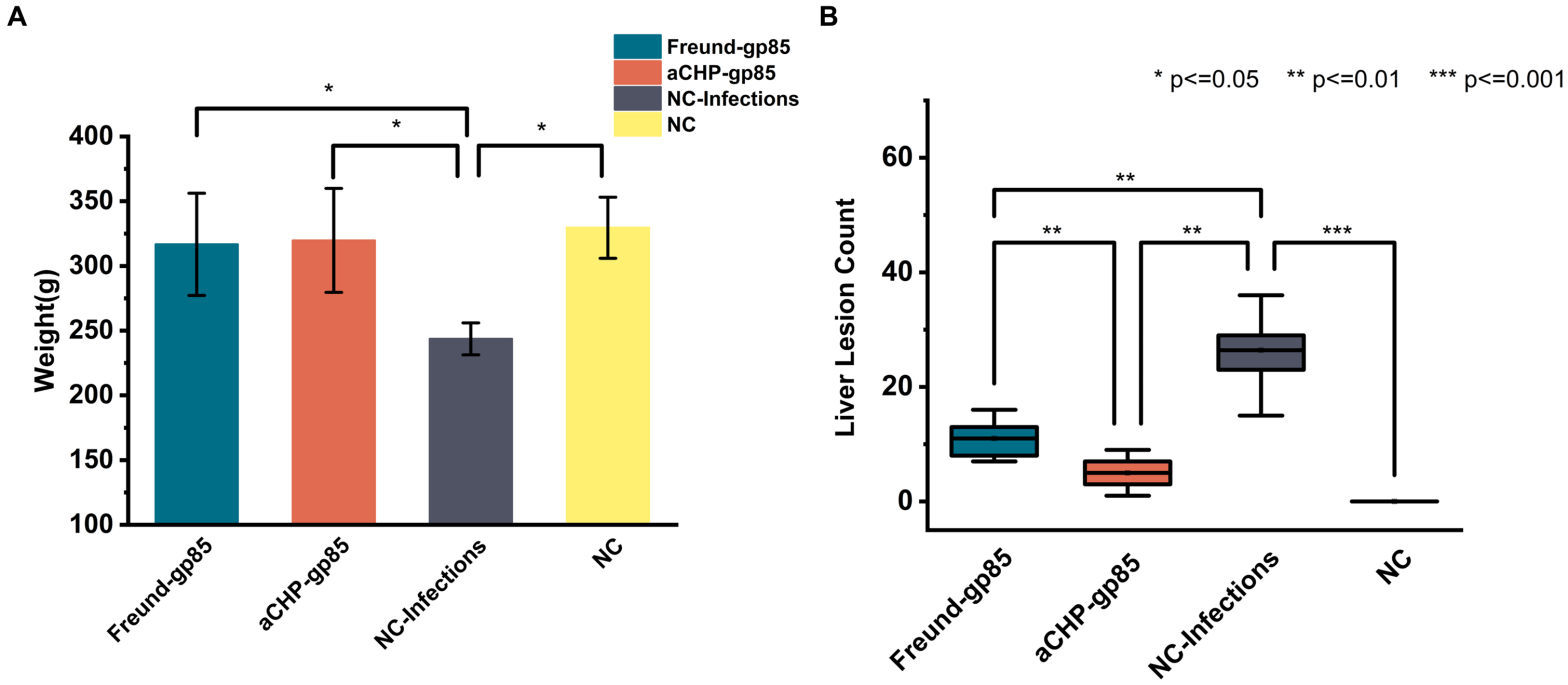
Weight and lesions of chickens in each group post-challenge. **(A)** Body weight comparison after challenge. Groups indicated. **(B)** Statistical comparison of liver lesion counts among chicken groups post-dissection (^*^indicates *p* < 0.05, ^**^indicates *p* < 0.01, and ^***^indicates *p* < 0.001).

Pre-dissection weight results, illustrated in Figure 8A, showed that both the aCHP-gp85 and Freund-gp85 groups, along with the NC group, had significantly higher body weights than the NC-Infections group. White lesions were noted in the livers of chickens in the NC-Infections and Freund-gp85 groups. However, the aCHP-gp85 group had significantly fewer lesions, indicating the vaccine’s effectiveness in partially halting disease progression, as highlighted in Figure 8B.

#### 3.6.4 Organ index differences

**Figure 9:**
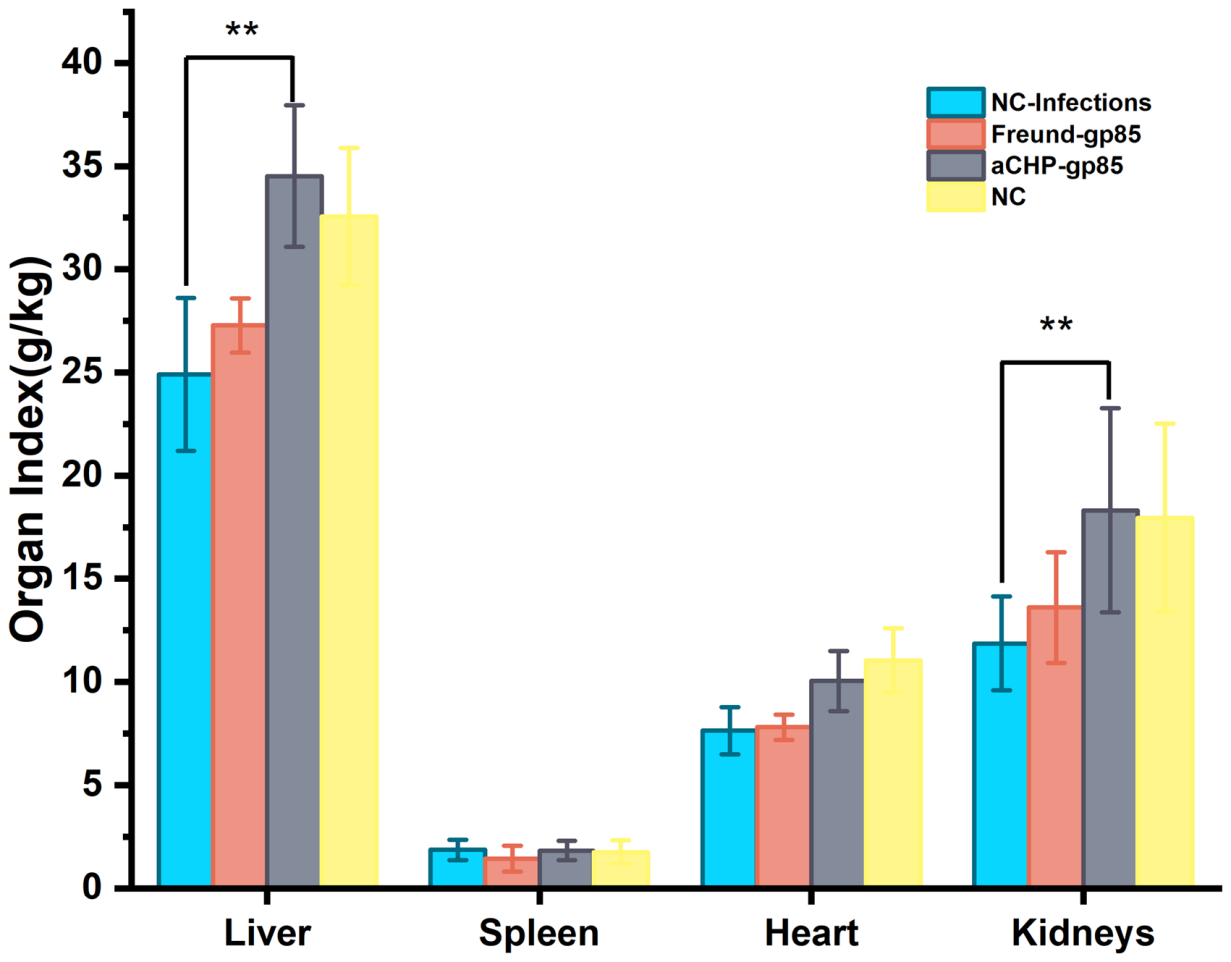
Organ index changes after the challenge. Organ indexes of chickens after the challenge. Organ index = weight of organ tissues/body weight of chicken × 100% (^*^indicates *p* < 0.05 and ^**^indicates *p* < 0.01).

Figure 9 displays the organ indices for each group of chickens. The NC-Infections group had significantly lower liver and kidney (*p* < 0.01) indices compared to the aCHP-gp85 group. There was no notable difference in organ indices between the Freund-gp85 and NC groups (*p* > 0.05).

#### 3.6.5 Comparison of organ viral load

**Figure 10:**
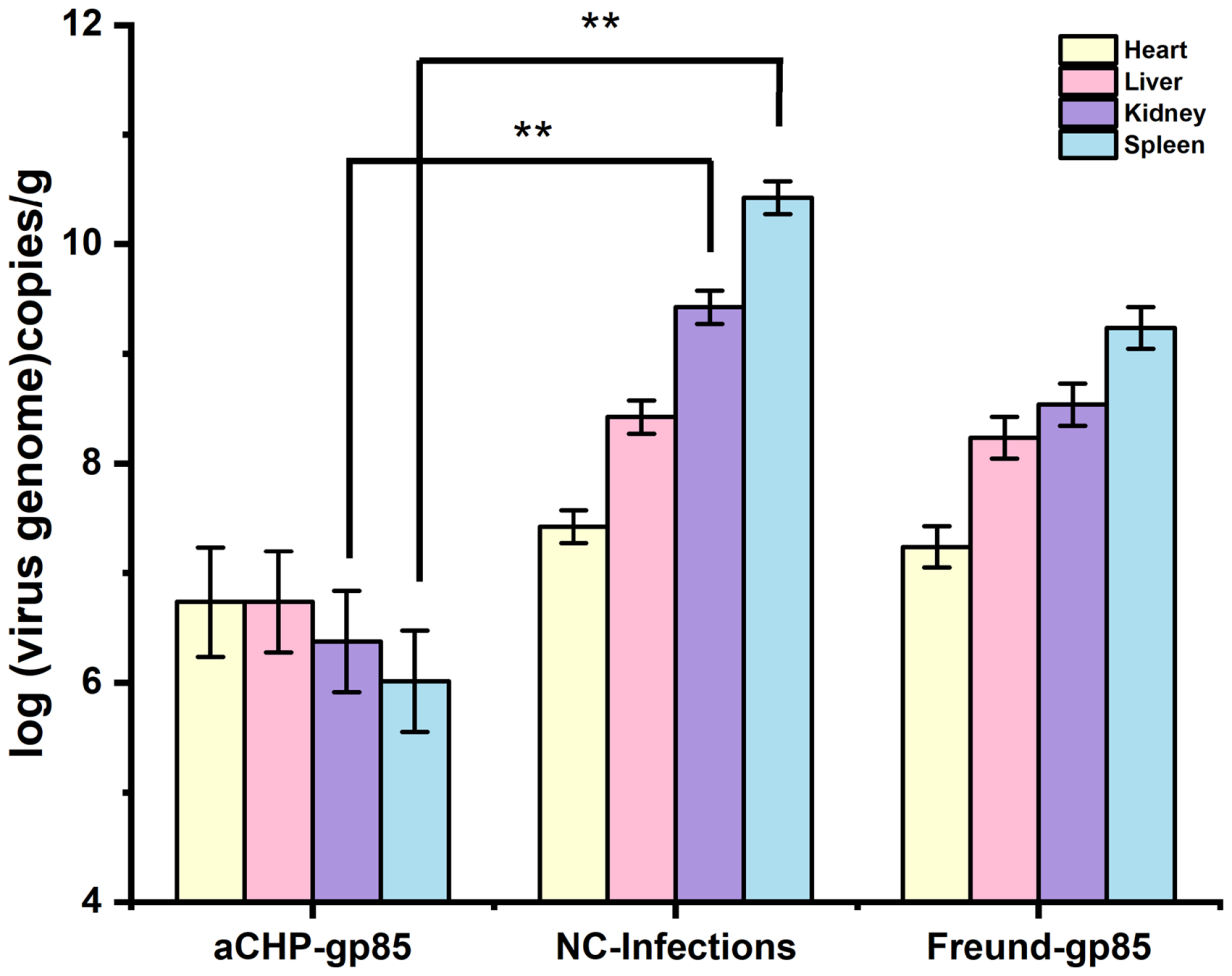
Viral load in chicken organs post-challenge. The viral load of chicken organs after the challenge. The virus was detected by fluorescence quantitative PCR, and each group of experiments was repeated three times (^*^indicates *p <* 0.05 and ^**^indicates *p <* 0.01).

The aCHP-gp85 group exhibited significantly lower viral loads in the kidney and spleen (*p* < 0.01), and slightly lower in the heart and liver (*p* < 0.01), compared to the NC-Infections group, as indicated in Figure 10. The Freund-gp85 group showed modest reductions in viral loads in the kidney and spleen relative to the NC-Infections group, but these were not statistically significant. These findings demonstrate that the aCHP-gp85 injection markedly reduced viral loads in the spleen and kidney, effectively controlling ALV-J.

## 4 Discussion

Previous research highlights the effectiveness of inactivated ALV vaccines in protecting specific chicken subpopulations (16). Vaccination increases the presence of ALV antibodies in breeding chickens, reducing the occurrence of diseases in infected birds and aiding in ALV management. The ALV-J gp85 protein, a key component of the viral envelope and the most variable structural protein of the ALV-J genome, is crucial for recognizing specific viral receptors on host cells. This specificity influences the virus’s subgroup and host range. Moreover, the gp85 protein plays a significant role in Li et al. (17) virus neutralization due to its high antigenicity, making it a common focus in ALV-J subunit vaccine research (18). This study confirms the ability of gp85 to induce a strong humoral immune response, which helps to reduce viremia and improve growth in chickens.

Biomacromolecular compounds, including sodium alginate, chitosan, and gums derived from plants, animals, fungi, and bacteria, have gained significant attention in the medical science community for their role in vaccine formulations and delivery systems (19). These compounds are valued for their non-toxicity, biocompatibility, biodegradability, and antiviral properties (20). Their application in enhancing vaccine safety and efficacy is well-documented (21, 22). This study focuses on the immunological effects of aCHP-gp85 and Freund-gp85. Our results show that aCHP-gp85 significantly boosts antibody levels against the gp85 protein over a longer duration than Freund-gp85. Notably, aCHP-gp85 induced higher IgG antibody levels in SPF chickens than vaccines using liposomes with recombinant gp85 protein vaccines (5). This heightened response is linked to chitosan’s ability to improve cellular and humoral immunity, enhancing antigen-presenting cell uptake due to its positive charge and interactions with negatively charged cell membranes. Additionally, it facilitates vaccine delivery through electrostatic interactions with DNA and negatively charged antigens (9). Alginate-chitosan composite microspheres hold promise in providing enhanced antigen protection, thereby prolonging the antigen’s efficacy within the chicken’s body.

Both the aCHP-gp85 and Freund-gp85 groups showed increased levels of mRNA transcription for immune markers, including IL-6, IL-1, TNF-α, IL-2, and IFN-γ, compared with the control group. The aCHP-gp85 group exhibited significantly higher TNF-α and IFN-γ mRNA transcription levels than the Freund-gp85 group (*p* < 0.05, *p* < 0.01), whereas IL-2 levels were notably lower in the aCHP-gp85 group (*p* < 0.01). For example, IFN-γ, an essential antiviral cytokine, boosts the production of Interferon-stimulated genes (ISGs) and diminishes ALV-J replication through the activation of antiviral proteins such as Mx, viperin, and IFITM3 (23). It further aids in T and B cell activation, NK cell differentiation and maturation, and prompts monocytes to release cytokines for immune response regulation (24). Conversely, TNF-α promotes macrophage and NK cell production, triggers inflammation, and thus helps in inhibiting tumor and viral replication (25). The stimulation of chicken splenic lymphocytes with ALV-J Gp85 protein significantly enhanced lymphocyte proliferation in the aCHP-gp85 group (*p* < 0.05), demonstrating its effectiveness in activating splenic lymphocytes for rapid proliferation and differentiation upon exposure to ALV-J Gp85 protein.

In challenge trials, aCHP-gp85 markedly reduced the ALV-J viral viremia rate, significantly improving growth in infected chickens. The growth retardation caused by ALV-J in chickens is associated with the suppression of the Wnt/β-catenin signaling pathway (26), suggesting the need for further studies on aCHP-gp85’s potential to activate this pathway. During necropsy, white nodules and splenomegaly were noted in the NC-Infections group, whereas white necrotic foci were observed in the Freund-gp85 group. By contrast, the aCHP-gp85 and NC groups showed no significant pathological changes. The organ index comparison revealed that aCHP-gp85 mitigated liver and kidney atrophy in infected chickens.

Analysis of viral loads in chicken organs post-inoculation showed that aCHP-gp85 significantly decreased viral loads in the spleen and kidneys, effectively limiting ALV-J spread. Chitosan’s role as an adjuvant, combined with its antibacterial and antiviral properties, offers a functional advantage over traditional adjuvants (27). Additionally, aCHP-gp85 presents promising research avenues in mucosal immunity, warranting further exploration in upcoming studies.

## Data availability statement

The raw data supporting the conclusions of this article will be made available by the authors, without undue reservation.

## Ethics statement

The animal study was approved by Ethics Committee of Longyan University (permit number: LY2021009x). The study was conducted in accordance with the local legislation and institutional requirements.

## Author contributions

TL: Conceptualization, Data curation, Formal analysis, Investigation, Methodology, Resources, Software, Validation, Visualization, Writing – original draft, Writing – review & editing. RL: Supervision, Validation, Writing – review & editing. LZ: Data curation, Investigation, Supervision, Validation, Writing – review & editing. TD: Investigation, Validation, Writing – review & editing. QM: Investigation, Validation, Writing – review & editing. XiaZ: Investigation, Validation, Writing – review & editing. YB: Resources, Writing – review & editing. CH: Funding acquisition, Resources, Writing – review & editing. WL: Funding acquisition, Resources, Writing – review & editing. YH: Resources, Writing – review & editing. XinZ: Conceptualization, Data curation, Formal analysis, Funding acquisition, Investigation, Methodology, Project administration, Resources, Supervision, Validation, Writing – review & editing.
